# Genome protection: histone H4 and beyond

**DOI:** 10.1007/s00294-020-01088-6

**Published:** 2020-06-17

**Authors:** Kundan Kumar, Romila Moirangthem, Rupinder Kaur

**Affiliations:** 1 Laboratory of Fungal Pathogenesis, Centre for DNA Fingerprinting and Diagnostics (CDFD), Hyderabad, Telangana 500039 India; 2 Graduate Studies, Manipal Academy of Higher Education, Manipal, Karnataka 576104 India

**Keywords:** Human fungal pathogens, Genome integrity, Homologous recombination, Methyl methanesulfonate (MMS), Stress resistance, Histones, Chromatin

## Abstract

Histone proteins regulate cellular factors’ accessibility to DNA, and histone dosage has previously been linked with DNA damage susceptibility and efficiency of DNA repair pathways. Surplus histones are known to impede the DNA repair process by interfering with the homologous recombination-mediated DNA repair in *Saccharomyces cerevisiae*. Here, we discuss the recent finding of association of methyl methanesulfonate (MMS) resistance with the reduced histone H4 gene dosage in the pathogenic yeast *Candida glabrata*. We have earlier shown that while the low histone H3 gene dosage led to MMS susceptibility, the lack of two H4-encoding ORFs, *CgHHF1* and *CgHHF2*, led to resistance to MMS-induced DNA damage. This resistance was linked with a higher rate of homologous recombination (HR). Taking these findings further, we review the interactome analysis of histones H3 and H4 in *C. glabrata*. We also report that the arginine residue present at the 95th position in the C-terminal tail of histone H4 protein is required for complementation of the MMS resistance in the *Cghhf1Δhhf2Δ* mutant, thereby pointing out a probable role of this residue in association with HR factors. Additionally, we present evidence that reduction in H4 protein levels may constitute an important part of varied stress responses in *C. glabrata*. Altogether, we present an overview of histone H4 dosage, HR-mediated repair of damaged DNA and stress resistance in this opportunistic human fungal pathogen.

## Introduction

Maintenance of genome integrity is pivotal to sustain life, with genome encountering regular threats from endogenous and exogenous stressors (Friedberg 2003; Ciccia and Elledge 2010). Genome stability is affected by various events including changes in the nucleotide sequence of DNA, single- and double-strand breaks in DNA, replication fork stalling and DNA–protein crosslinks (Lindahl 1993; Tretyakova et al. 2015). These alterations in DNA are sensed, signaled, and subsequently repaired by a large repertoire of proteins whose subcellular localization, activity and functions are exquisitely coordinated (Friedberg 2003; Ciccia and Elledge 2010). It is, therefore, not surprising that the genome integrity is closely entwined with regulation of the cell cycle progression (Novák et al. 2018). However, an unaltered genome is a double-edged sword. While on the one hand, faithful replication and transmission of a stable genome across generations is important to maintain life, evolution of a new species, on the other hand, is predominantly governed by genetic variations (Hurles 2005; Charlesworth and Charlesworth 2009; Bateson 2017). These alterations in the genome may assist in rapid adaptation to changing environmental conditions and confer survival advantage (Brooks et al. 2011; Bateson 2017). A delicate balance, therefore, is often maintained between genome stability and variability rate (Hurles 2005; Lynch 2010; Brooks et al. 2011; Croll and McDonald 2012; Bateson 2017). *Candida glabrata* is an opportunistic human fungal pathogen, with a haploid genome (Dujon et al. 2004; Gabaldón and Fairhead 2019; Kumar et al. 2019), whose genome dynamic mechanisms are yet to be characterized. It shares a common ancestor possessing a duplicated genome with the budding yeast *Saccharomyces cerevisiae*, with both yeasts undergoing massive gene loss after divergence (Dujon et al. 2004). Comparative genome analyses have pointed towards the recent origin, from an evolutionary standpoint, of the ability of *C. glabrata* to infect humans, with homologous recombination (HR) and rearrangements in subtelomeric regions of the genome contributing to antigenic plasticity (Gabaldón and Fairhead 2019; Juárez-Reyes and Castaño 2019). In this Perspective article, we focus on the nexus among histone H4 dosage, HR efficiency and resistance to DNA damage in *C. glabrata*.

## Histones and DNA damage

Genome in eukaryotes is packaged into a dynamic macromolecular structure, chromatin, whose basic structural unit is Nucleosome (Campos and Reinberg 2009; Hauer and Gasser 2017), with euchromatin (transcriptionally active) and heterochromatin (transcriptionally silent) containing low and high density of nucleosomes, respectively (Janssen et al. 2018). Telomeric and centromeric heterochromatin contribute to genomic stability by suppressing recombination between homologous subtelomeric sequences, and transcriptional silencing of pericentromeric repeats, respectively (Janssen et al. 2018; Greenstein and Al-Sady 2019; Nakagawa and Okita 2019). A nucleosome is made up of 146 bp long DNA wrapped around an octamer of histone proteins (two molecules each of four core histone proteins, H2A, H2B, H3 and H4) (Kornberg and Thonmas 1974; Campos and Reinberg 2009; Hauer and Gasser 2017). Histones are highly conserved, basic proteins which regulate a wide range of DNA metabolic processes including replication, recombination, repair and transcription (Campos and Reinberg 2009; Hauer and Gasser 2017). Histone genes are encoded by multi-copy gene families which ensure regulated supply of histones during both favourable and adverse conditions (Hentschel and Birnstiel 1981; Campos and Reinberg 2009; Kurat et al. 2014). Alterations in histone levels, posttranslational modifications (PTMs) and protein–protein interactions modulate chromatin structure and functions (Peterson and Laniel 2004; Gunjan et al. 2006; Campos and Reinberg 2009; Singh et al. 2010; Hauer and Gasser 2017; Ichikawa and Kaufman 2019).

The levels of histones H3 and H4, which are present in both free and chromatin-bound form, are tightly regulated, due to deleterious consequences of histone overexpression (Gunjan et al. 2006; Singh et al. 2010). Histone H3 and H4 biosynthesis occurs during S phase of the cell cycle, and the cellular response to genotoxic insults involves a wholesale reduction in levels of core histones, which is achieved by both transcriptional repression of histone genes and proteasome-mediated degradation of histone proteins (Gasch et al. 2001; Su et al. 2004; Gunjan et al. 2006; Singh et al. 2010; Kurat et al. 2014; Hauer et al. 2017). In *S. cerevisiae*, both chromatin-bound (Hauer et al. 2017) and free-pools of H3 and H4 (Liang et al. 2012) are degraded upon DNA damage. Further, deletion of one of the two gene copies of H3 and H4, and partial depletion of H4 are known to lead to MMS (methyl methanesulfonate; DNA alkylating agent) resistance, and genomic instability due to increased HR, respectively (Prado and Aguilera 2005; Liang et al. 2012).

Recently, MMS exposure was found to cause significant reduction in mRNA and protein amounts of both H3 and H4 histones in the pathogenic yeast *C. glabrata*, with low H4 gene dosage also giving rise to MMS resistance (Kumar et al. 2020). In this study, we had shown a requirement for HR in the repair of MMS-induced DNA damage, and low H4 levels resulting in increased HR efficiency, faster repair of damaged DNA and MMS resistance (Kumar et al. 2020). However, this MMS resistance was specific to the loss of two H4-encoding genes, probably due to the H4 amount produced by the remaining H4 ORF (Kumar et al. 2020). *C. glabrata* contains three histone H4 genes, *CgHHF1, 2* and *3*, with *CgHHF1* and* 2* genes exhibiting synteny with their *S. cerevisiae* counterparts (Kumar et al. 2020). Of these, while *CgHHF1* and* 2* gene loss rendered *C. glabrata* cells resistant to MMS, deletion of *CgHHF2* and* 3* genes made cells sensitive to MMS (Kumar et al. 2020), indicating that the deletion of two H4 genes can have opposite biological consequences. Notably, MMS resistance was not observed in any mutant carrying low dosage of histone H3 genes (Kumar et al. 2020). Furthermore, reduction in the H4 gene dosage had no significant impact on the transcript abundance of histone H3, while the converse was not always true (Kumar et al. 2020). These findings point towards plausible disparate regulatory mechanisms for H3 and H4 expression in the pathogenic yeast *C. glabrata*, and underscore a negative role for H4 in HR pathway.

## Histone H4 and homologous recombination in *C. glabrata*

Histone H3 and H4 genes are usually present in a paired form, with shared regulatory regions, and H3 and H4 proteins form a heterotetramer, which constitutes the central core of the histone octamer in the nucleosome (Hentschel and Birnstiel 1981; Peterson and Laniel 2004; Campos and Reinberg 2009; Kurat et al. 2014; Hauer and Gasser 2017). HR plays an important role in DNA damage repair, and chromatin dynamics during DNA repair includes PTMs and displacement of H3 and H4, and disassembly and reassembly of nucleosomes (Bishop and Schiestl 2000; Hauer et al. 2017; Hauer and Gasser 2017). Consistently, histones H3 and H4 have been implicated in homologous recombination, with these proteins also interacting physically with HR factors, and competing with HR components for binding to damaged DNA in *S. cerevisiae* (Liang et al. 2012).

This notwithstanding, the protein interactome of H3 and H4 are not identical. In *S. cerevisiae*, H3 and H4 have 452 common interacting proteins, which represent 58% of the total H4 interactome (https://www.thebiogrid.org/). Consistently, we reported in *C. glabrata* an overlap of 89 proteins, constituting 53% of the H4 interactome, between the interactome of H3 and H4 under regular growth conditions (Kumar et al. 2020). Furthermore, MMS treatment drastically reduced and slightly increased the protein partners of histone H4 and H3, respectively, in *C. glabrata*, with H3 and H4 sharing nine common protein interactors under this DNA-damaging condition (Kumar et al. 2020). These data point towards distinct and intricate regulation of protein–protein interaction for two of the core histone proteins in *C. glabrata*, and raise the question of this regulation being unique to pathogenic fungi. In this context, it is worth noting that compared to *S. cerevisiae*, *C. glabrata* has higher rate of non-homologous end-joining recombination (Cormack and Falkow 1999; Corrigan et al. 2013). A careful analysis revealed only one HR pathway protein CgRfa2 (subunit of heterotrimeric replication protein A) to be present in the *C. glabrata* H4 interactome under regular growth conditions (Kumar et al. 2020). Notably, the *S. cerevisiae* H4 interactome contained 6 HR pathway proteins, including Rfa2, with 66 proteins being common H4 interactors in both yeasts (https://www.thebiogrid.org/) (Kumar et al. 2020). This may signify that H4 either does not directly interact with HR factors under regular growth conditions, or H4-HR component interaction is transient in *C. glabrata*. Whether differences in the type of H4 interactors between *C. glabrata* and *S. cerevisiae* have any correlation with the number (2 in *S. cerevisiae* and 3 in *C. glabrata*) and regulation of H4-encoding genes, and/or resultant varied H4 protein levels, remains to be determined. Lastly, although MMS sensitivity phenotype of the mutant lacking two histone H3 genes (Kumar et al. 2020) suggests no increase in HR frequency in this mutant, the effect of histone H3 on HR in *C. glabrata* is yet to be demonstrated.

**Fig. 1:**
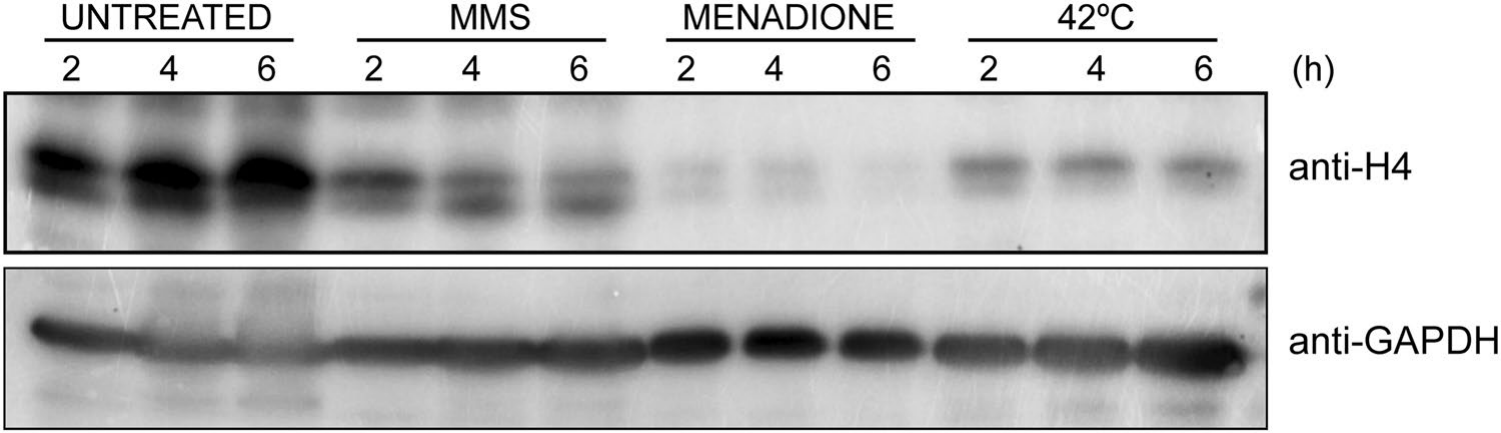
Histone H4 levels are reduced in *C. glabrata* upon exposure to thermal and oxidative stresses. Representative immunoblot showing histone H4 levels in *C. glabrata wild-type* (*wt*) cells under indicated growth conditions. *wt* cells were grown to logarithmic phase in YPD medium, and either left untreated or treated with 0.03% MMS or 100 µM menadione for indicated time intervals at 30 °C. For thermal stress, cells were grown at 42 °C in YPD medium. Cells were collected at indicated time points and lysed using glass beads. Cell lysates (50 µg protein) were resolved on 15% SDS-PAGE and probed with anti-H4 and anti-GAPDH antibodies. CgGapdh was used as a loading control

Under what conditions, does *C. glabrata* downregulate histone H4 levels? Our analysis showed a decrease in H4 levels upon growth at 42 °C (thermal stress) and in the presence of menadione (oxidative stress) (Fig. 1), in addition to, upon macrophage internalization and MMS exposure, as reported previously (Rai et al. 2012; Kumar et al. 2020). This reflects that reduction in H4 levels in *C. glabrata* could be a general stress response, and may aid in survival of the hostile host environment. Of note, reduction in histone mRNA in mammalian cells has recently been linked with a better recovery from apoptosis (Tang et al. 2017). Whether the reduced H4 amounts in *C. glabrata* free up proteins required to survive stress, or transcriptionally activate the expression of stress proteins are possibilities worth-testing.

## The arginine-95 amino acid residue in the C-terminal tail of H4 is required for modulation of MMS tolerance

**Fig. 2:**
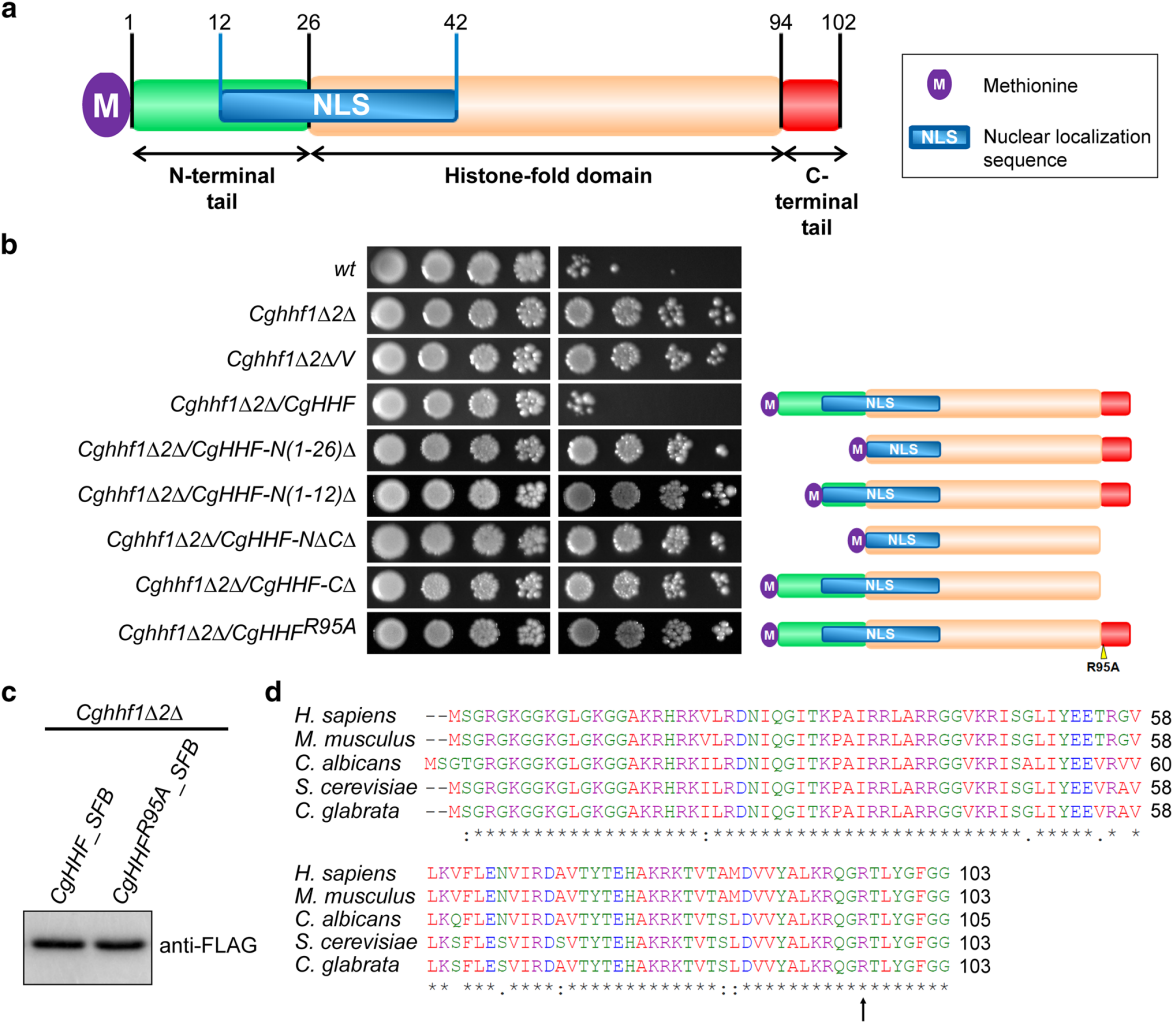
The arginine-95 residue in histone H4 is required for modulation of MMS susceptibility. **a** A schematic illustration of domain organization of the *S. cerevisiae* Hhf (histone H4) protein. Histone H4 has a central histone-fold domain flanked by a long N-terminal tail (26 aa) and a short C-terminal tail (8 aa). One bipartite NLS (31 aa), spanning the N-terminal tail and the histone-fold domain, as predicted by the NLS mapper tool (https://www.nls-mapper.iab.keio.ac.jp), is also shown. **b** Serial dilution spot assay showing a requirement for arginine-95 for reversal of MMS resistance in the *Cghhf1Δ2Δ* mutant. Indicated *C. glabrata* cultures expressing full-length histone H4, H4 lacking different regions or H4 carrying alanine in place of arginine at 95th position, were grown overnight in CAA medium, normalized to an OD_600_ of 1.0, and tenfold serially diluted in PBS. 3 µl of each dilution was spotted on YPD medium lacking or containing 0.06% MMS, and plates were incubated at 30 °C. Plates were photographed after day 2 for YPD, and day 3 for 0.06% MMS. Schematic representation of each construct is shown on the right side of the spot image. **c** An immunoblot showing expression of SFB (triple epitope)-tagged histone H4 and H4^*R95A*^. The *Cghhf1Δ2Δ* mutant expressing *CgHHF-SFB* or *CgHHF*^*R95A*^*-SFB* were grown in CAA medium for 4 h at 30 °C to get mid-log phase culture. Post incubation, cells were harvested and whole-cell extracts were prepared by glass bead lysis. 50 µg protein were resolved on 15% SDS-PAGE and probed with anti-FLAG antibody. A band of 27 kDa, corresponding to H4-SFB, was seen in both samples. **d** Amino acid sequence alignment of the histone H4 protein of *C. glabrata, C. albicans, S. cerevisiae, Mus musculus* and *Homo sapiens*, showing highly conserved C-terminal tail. Histone H4 protein sequences were taken from Candida genome database (CGD), Saccharomyces genome database (SGD) and Uniprot database, and aligned using the Clustal Omega multiple sequence alignment tool (https://www.ebi.ac.uk/Tools/msa/clustalo/). The black asterisk indicates the identical amino acid residue. Arginine-95 is marked by an arrow. Please note that serine is considered as the first amino acid of the histone H4 protein, due to the excision of the initial methionine residue

The histone H4 protein contains a core histone-fold domain and unstructured amino (N)- and carboxy (C)-terminal tails (Luger et al. 1997). To address the question of which part of histone H4 modulates MMS resistance in *C. glabrata*, we carried out the domain-deletion analysis for H4. The histone H4 protein in *C. glabrata* consists of 103 amino acid residues, with its N-terminus carrying a canonical nuclear localization signal (NLS) (Fig. 2a). We created five H4 protein variants that lacked either N-terminal and/or C-terminal region of H4. We found that neither a large (26 aa region) nor a small (12 aa region) deletion in the N-terminal region of histone H4 could render *Cghhf1Δhhf2Δ* cells susceptible to MMS, upon ectopic expression (Fig. 2b). Similarly, the C-terminal tail, and particularly the arginine residue at 95th position (R-95), were also required for MMS tolerance, as *Cghhf1Δhhf2Δ* cells expressing histone H4 either carrying alanine substitution of arginine-95 residue or without the terminal eight amino acids, could grow well in MMS-containing medium (Fig. 2b). This functional loss was not associated with reduced expression, as CgHhf^*R95A*^ (histone H4^*R95A*^) protein was expressed in good amount in *C. glabrata* cells (Fig. 2c). Altogether, these data suggest that both N- and C-termini of H4 are required for MMS susceptibility modulation, and by extension, efficient HR system.

The arginine-95 residue, though conserved among histone H4 of *C. glabrata, S. cerevisiae, C. albicans,* mice and humans (Fig. 2d), is yet to be assigned any function or a modification. Of note, the C-terminal tail of *S. cerevisiae* H4 has been reported to be flexible, and this flexibility was required for nucleosome remodelling (Chavez et al. 2012). The C-terminal H4 tail also interacts with Asf1 chaperone, and serves as a lever to promote Asf1-dependent chromatin organization (English et al. 2006). Interestingly, alanine substitutions of any of the three adjacent amino acids, lysine-97, tyrosine-98, or glycine-99, in the C-terminus of *S. cerevisiae* H4, led to polyploidy (Yu et al. 2011). Altogether, these results highlight the importance of the C-terminal region of the histone H4 in maintenance of the chromatin architecture and genome stability. Since *C. glabrata* and *S. cerevisiae* H4 proteins are identical in amino acid sequence, but for one amino acid at the 69th position (Fig. 2d), future studies will investigate whether the C-terminal tail of *C. glabrata* H4 interacts with histone chaperones, and/or the arginine-95 residue is subjected to posttranslational modifications in *C. glabrata*, which may govern histone H4 levels, that in turn will modulate HR efficiency. In this context, it is worth noting that methylation at arginine-3 residue in H4 has recently been reported to modulate cellular senescence in human cells by regulating H4 protein stability (Lin et al. 2020).

## Concluding remarks

The histone H4 levels play an important role in maintaining genome stability via regulating homologous recombination, as reduced histone H4 dosage was found to be linked with resistance to MMS-induced DNA damage and higher rate of HR in the pathogenic yeast *C. glabrata*. Since HR ensures high-fidelity repair of damaged DNA, increased HR is particularly advantageous when cells have to cope with high degree of DNA damage, and stable genome maintenance is of utmost priority. *C. glabrata* probably achieves this by downregulating histone H4 levels. The current findings raise several questions, including the significance of H3 amount reduction for MMS stress survival, role of arginine-95 in H4 interaction with HR factors, and most importantly the impact of H4-modulated HR on fungal virulence. Since the *Cghhf1Δhhf2Δ* mutant showed no survival defect in the murine model of systemic candidiasis (Kumar et al. 2020), further studies are warranted to investigate if elevated HR confers a distinct advantage to *C. glabrata* in a discrete host environmental niche.
